# Probing Transferrin Receptor Overexpression in Gastric Cancer Mice Models

**DOI:** 10.1021/acsomega.1c04382

**Published:** 2021-10-27

**Authors:** Madeeha Shahzad Lodhi, Muhammad Tahir Khan, Syed Mulazim Hussain Bukhari, Sajjad Hussain Sabir, Zahoor Qadir Samra, Haider Butt, Muhammad Safwan Akram

**Affiliations:** † Institute of Molecular Biology and Biotechnology (IMBB), The University of Lahore, KM Defence Road, Lahore 58810, Pakistan; ‡ Azad Jammu Kashmir Medical College, Stadium Road, Jalalabad, Muzaffarabad 13100, Pakistan; § Department of Gastroenterology and Hepatology GHAQ Teaching Hospital, Sahiwal 57000, Pakistan; ∥ Institute of Biochemistry and Biotechnology, University of the Punjab, Lahore 54590, Pakistan; ⊥ Department of Mechanical Engineering, Khalifa University, P.O. Box 127788, Abu Dhabi 23667, UAE; # School of Science &amp; Health, Teesside University, Middlesbrough TS1 3BA, U.K.; ∇ National Horizons Centre, Teesside University, 38 John Dixon Ln, Darlington DL1 1HG, U.K.

## Abstract

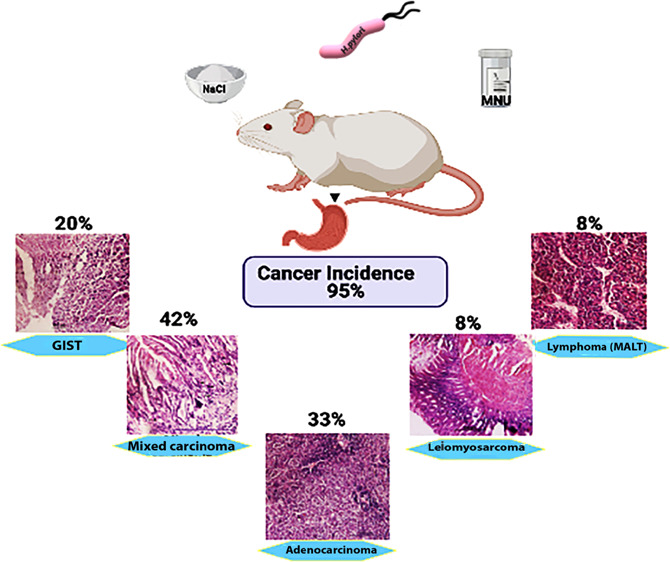

Exposure to carcinogenic chemicals, *Helicobacter pylori* infection, and high dietary salt are the risk factors associated with gastric cancer. Mice models of gastric cancer are key to understanding the cancer mechanism, to discerning the role played by different factors, and to determining therapeutic effects of different treatments. The goal has been to find targets which are only expressed with cancer so that they can be targeted specifically without harming normal cells. One such target could be the transferrin receptor, a glycoprotein receptor that is expressed many-folds on rapidly growing cells due to the greater demand of iron. In this study, gastric cancer was developed in mice (BALB/c) with human cancer-associated risk factors by feeding them with tumor-inducing concentration of methyl nitrosourea, dietary salt, and *H. pylori* along with normal feed and water. Three strategies were adopted to induce gastric cancer; (1) use of *N*-methyl-*N*-nitrosourea (MNU) with high dietary salt (NaCl), (2) infection with *H. pylori* (isolated from human gastric tissue), and (3) use of MNU along with high concentration of NaCl after *H. pylori* infection. Mice were dissected after induction, and histological study of gastric tissue was done with Hematoxylin and Eosin staining. A diagnostic probe comprising transferrin conjugated with cadmium sulfide quantum dots was prepared and characterized. It was used to study the transferrin receptor overexpression in gastric tissue of cancer-induced mice relative to the normal mice. Mice of group 3 showed the highest rate of the cancer incidence ratio (96%) along with a high expression of transferrin receptors among the three groups. Histochemical studies showed that different types of gastric cancer depend upon the cancer-induction conditions. The mouse model of group 3 has the potential to be used in the future to study the therapeutic effects of cancer medicines, and overexpression of transferrin receptors could be identified through the designed probe to be used as diagnostics.

## 1 Introduction

Among all dreadful diseases, cancer is the foremost cause of death around the world, and its incidence is increasing worldwide. Gastric cancer is its most prevalent type worldwide with high mortality, causing around one million deaths worldwide.^1^ In Asia, it is the second most common cause of death,^2^ and to detect it early is the only way to reduce the mortality and burden of the disease. Gastric carcinoma may be due to many factors such as carcinogenic chemicals including high salt intake, organic solvents, insecticides, pesticides, microbial infections, e.g., *Helicobacter pylori*,^3^ and occurrence of certain mutations.^4^ In all the cases, it appears to originate from the lining of the stomach. The expression of transferrin receptors increases many-folds on cancer cells as compared to normal cells.^5,6^ This positive correlation^7^ can be justified due to the high iron demand of rapidly growing cells.^8,9^ This relationship makes the transferrin receptor an important target for drug delivery of nanomedicines either through transferrin protein^10,11^ or using antitransferrin receptor antibodies.^12^ Such targeted drug delivery can potentially avoid the toxic effects on normal cells and allow for a limited concentration of drug to be released in a controlled manner.^13^

Animal models enhance our understanding of various cancers at the molecular level and also help us to check the therapeutic effects of newly designed drugs prior to human trials.^14,15^ In this study, a mouse model is established to study the effects of *H. pylori,* chemical carcinogens (*N*-methyl-*N*′-nitro-*N*-nitrosoguanidine (MNNG) and *N*-methyl-*N*-nitrosourea (MNU)), or by using the combination of both chemicals and microbes in the induction of gastric cancer. BALB/c mice were used to develop the gastric cancer model as they have a very well characterized disease pathology. The particular strain has established reticular, lung, and renal tumors but has been shown to be resistant to develop colon cancer.^16,17^ Therefore, a proper strategy is required to develop the BALB/c gastric cancer model.

*H. pylori* has a significant effect on stomach pathogenesis and etiology. The need for an animal model to understand human gastritis and cancer is strongly reflected in the literature.^18^ Studies have reported different levels of gastric pathology in different animal species with prolonged *H. pylori* infection.^15^ Similarly, animal models of chemical carcinogenesis are developed to study the effect of different chemicals in gastric pathology. Compounds containing nitroso functional groups such as nitroso amines, e.g., MNNG and MNU are considered to be an important gastric cancer-inducing agent in humans (generated by anaerobic bacteria^19^). However, lack of chronic inflammation (caused by *H. pylori* infection) in chemically induced gastric cancers have been a drawback of such models. Therefore, in this study we have combined both factors to have a more realistic model of the actual disease.^19^ This model will have applications in studying the therapeutic effects of new medicines. This study is designed for the early detection of cancer by probing the transferrin receptor overexpression. For this, we have used transferring-coated cadmium sulfide quantum dots (QDs), which have myriad applications in biosciences due to their unique optical, electronic, and thermal properties. They have a high energy band gap and their surface chemistry can easily be manipulated by capping with useful functional groups and linkers, which can act as stabilizing agents and can be used for conjugation with ligands and medicines.^20,21^ Effects and induction levels of the mentioned cancer-causing agents were correlated with relative expression of transferrin receptors on gastric tissue in order to probe the cancerous tissue for the potential future treatment.

## 2 Results and Discussion

Mice models are reliable for studying the exposure effects of different cancer-associated risk factors for various cancers and allow us to design and test therapeutics against them.^15^ A number of procedures have been adopted to induce gastric cancer in mice including the exposure to MNU, high dietary salt concentration, prolonged infection with *H. pylori*, and genetically induced gastric cancer.^15,22^ The main objective of the study is to find targeted sites of rapidly dividing cells in the mouse model of gastric cancer by probing transferrin receptors, expression of which was examined in affected gastric tissues of all mice groups with different pathological severities. In order to get an effective response from the mouse model that closely showed the human gastric cancer physiology, three cancer-induction methods were designed as described in the Section 4. The long-term exposure with carcinogenic chemicals and *H. pylori* for cancer induction has increased the cancer incidence ratio with discernible stomach pathology. This study is not only useful to establish a successful gastric cancer model in BALB/c mice but also useful to correlate the transferrin receptor expression with cancer pathology. Future application of this work includes the use of the modified QDs and nano/microparticles as a vehicle for drug delivery to cancer cells.

### 2.1 Group 1(MNU and NaCl-Induced Cancer)

A total of 50 mice (25 males/25 females) were housed under this group, 20 mice for a 6 month study and 30 mice for 12 months. One male in the 6 month group died after 3 weeks and was excluded from the study. After 26 weeks of exposure to MNU + NaCl cancer induction, 19 mice were dissected. In the 12 month induction group, five mice died before 40 weeks and therefore were excluded from the study (total 25) while two mice died after 45 weeks but were retained in the study. Mice were dissected at predetermined times and blood and stomach samples were collected for serology, histology, and immunohistochemical studies. The average initial weight of the animals was 20 ± 2 g and a gradual gain of weight was observed up to 15 weeks. After 15 weeks animals showed variation in gaining weight according to the level of severity of affected stomach tissues. After 40 weeks of inoculation, mice became lethargic and started to lose weight.

**Figure 1:**
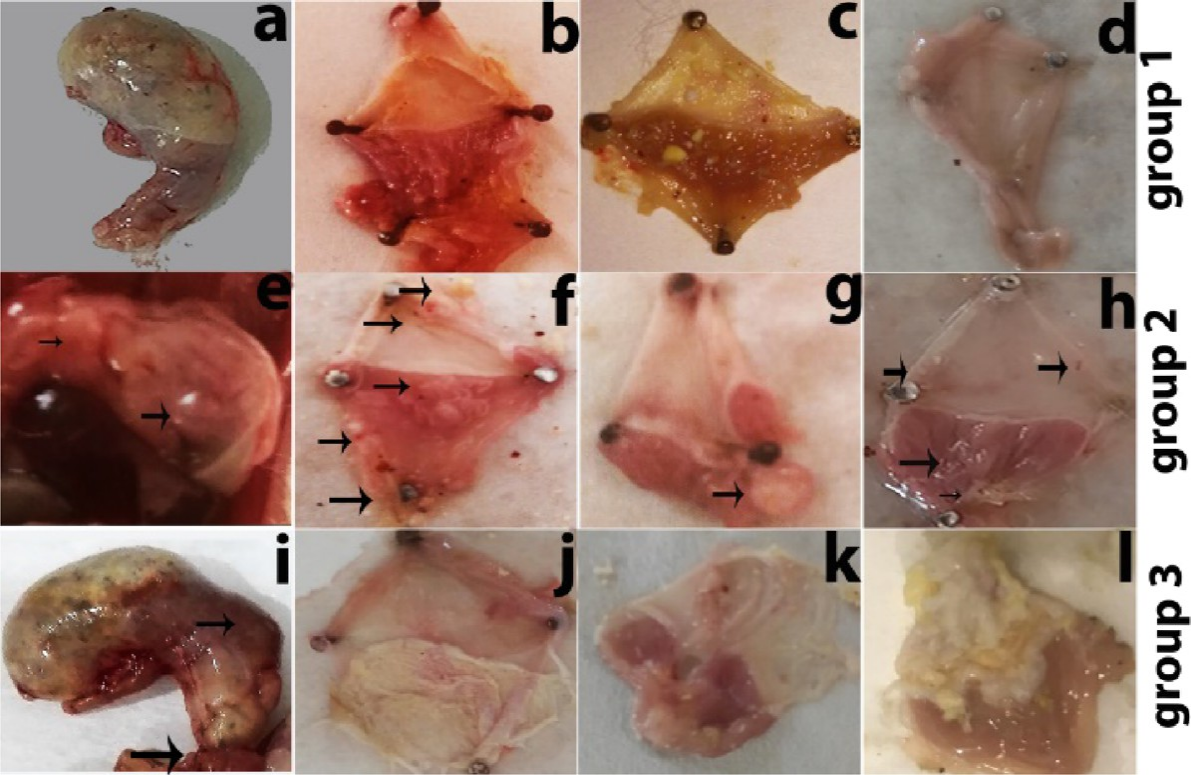
Macroscopic examination of the stomach of groups 1, 2, and 3 mice. Gross anatomical studies of the infected stomach show inflammation and protruded masses at the stomach outer surfaces with severe infection in group 1 and 2 mice (a and e). The opened stomach (from the lesser curvature) shows a change in color due to hemorrhage with an uneven surface, inflammation, lesions, abscesses and a number of small polyps (b and c), and severe changes in morphology with abnormal surfaces (d). Open stomach examinations of group 2 mice show uneven surfaces, change in coloration, abscess, inflammation, lesion, and polypoid masses, indicated by arrows (f, g, and h). The gross anatomy of the group 3 mouse stomach with abnormal surface pathology, protruded masses, change in color, tumor, and inflammation (i). The morphological study of the group 3 mouse open stomach shows a change in coloration, abscess, inflammation, lesions (j), tumor masses and a severely affected surface (k), and sarcoma in polyps (l).

The gross anatomy of the stomach was observed, and it showed that the stomach lining was uneven with lesions and abscesses. Several small polyp masses were observed in mice treated with MNU and high diet salt for 6 months. Surface morphology of the mouse group after 12 months was more severe, showing abnormal surface pathology with protruded masses, uneven surface, hemorrhage, inflammation, abscesses, polyps, and small tumors (Figure 1a–d).

**Figure 2:**
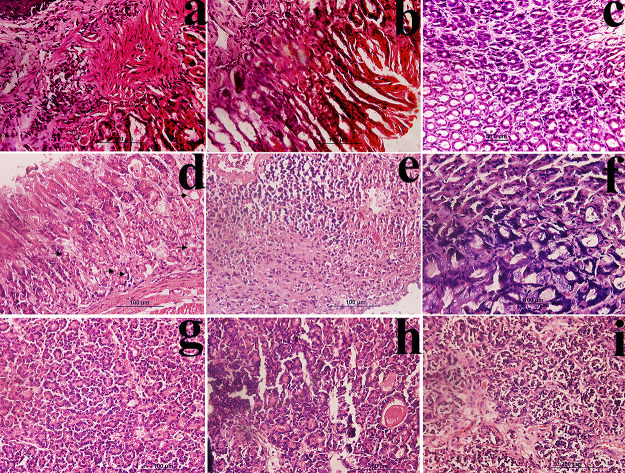
MNU+ high salt diet-induced gastric carcinoma (H&E 40×). After 6 months: Photomicrographs show (a) tumor desmoplasia with invasive adenocarcinoma and mucinous infiltration with inflammatory cells. (b) Neoplastic lesion with carcinoma in situ and superficial atypia. After 12 months: Photomicrographs show (c) hyperplasic conditions with dysplastic changes and fusion of cells. (d) Mixed type of carcinoma polypoidal with signet ring cell carcinoma (indicated by arrows). (e) Morphological changes in gastric tissue due to GIST. Histological sections reveal spindle cells with marked atypia, hyperchromasia, and malignant gastrointestinal tumor. (f) Adenocarcinoma with the presence of giant tumor cells. (g) Well-differentiated adenocarcinoma, (h) moderately differentiated adenocarcinoma with mucosal infiltration of inflammatory cells, and (i) tumor desmoplasia with invasive adenocarcinoma and mucinous infiltration with inflammatory cells.

Histological examination (after 6 months) (Figure 2a,b) showed severe gastritis, atrophy, and in some cases dysplasia along with inflammatory response, invasive carcinoma, and adenocarcinoma. Epithelial layer erosion, epithelial cell abnormalities, hypertrophy, and hyperplasia with fusion of the cells were the most common conditions prevailing in this group. Cross-section studies showed that most of the animal’s tissue had polypoidal dysplastic lesions, hemorrhage, neoplastic lesion, influx of inflammatory cells in whole stomach tissue, and mucinous abnormalities.

Histological study (after 12 months) showed severe inflammation with influx of neutrophils and monocytes in mucus cells and the mucinous layer. Inflammation also resulted in the loss of parietal cells that led to dysplasia in some cases (Figure 2c). Histological sections reveal spindle cells with marked atypia, hyperchromasia, and malignant gastrointestinal tumor (GIST) as shown in Figure 2e. The study also revealed the presence of a mixed type of cancer in mice like polypoidal with signet ring cell carcinoma (Figure 2d), adenocarcinoma with signet rings, and diffused carcinoma with signet ring cell carcinoma. Most of the histological sections showed the dysplastic changes with the influx of inflammatory cells and the presence of giant cells and tumor cells (Figure 2f). Different forms and stages of cancer cells were studied, like tumor desmoplasia with invasive adenocarcinoma (Figure 2i), well differentiated adenocarcinoma (Figure 2g), and moderately differentiated (Figure 2h) and poorly differentiated tumor carcinoma.

Table 1 shows the gastric pathology and incidence of gastric cancer that is 36% after 6 months and 68% after 12 months of treatment for cancer induction based on histological study results in group 1. Inflammation and abscess were observed in 100% mice. Induced cancer included gastrointestinal stromal cell carcinoma, signet ring cell carcinoma, polypoidal adenocarcinoma, and neuroendocrine gastric tumor carcinoma.

**Table 1:** Gross Anatomy and Histopathological Findings of the Stomach after 6 and 12 Months of Study in Groups 1, 2, and 3^a^

	group 1		group 2		group 3	
findings	6 m	12 m	6 m	12 m	6 m	12 m
total number of animals	20	30	25	20	30	30
dead during infection	1	5	5	4	7	11
cancer incidence percentage	36%	68%	25%	55%	65%	96%
uneven surface and hemorrhage	10	25	4	16	17	24
presence of inflammatory cells	17	25	20	16	23	24
mucosal and submucosal abscess	19	25	15	16	23	24
GIST	0	2	0	1	3	5
signet ring cell carcinoma	0	3	0	1	1	3
polypoid adenocarcinoma	1	2	0	0	3	6
metaplasia	0	0	3	4	2	5
dysplasia	4	3	2	5	8	10
sarcoma	0	1	0	0	0	2
adenocarcinoma	2	7	0	1	4	8
lymphoma	0	0	0	2	0	2
mixed carcinoma	1	4	0	2	2	10

a Some mice showed mixed carcinoma; therefore, they are counted in more than one column of the existing cancer type.

Group 1 results are in agreement with the previously published studies in different animal species and in different mouse species, though the cancer/inflammation incident rate is different.^3^*N*-Methyl-*N*-nitrosourea (MNU) was also used to promote gastric carcinogenesis in wild-type (wt) and *cdh1^+/–^* mice and reported 75% tumor incidence in the selected species.^23^

### 2.2 Group 2 (*H. pylori-*Induced Cancer)

*H. pylori* is Gram-negative bacteria that colonize the gastric epithelial cells and infect almost half of the world population. Gastric *H. pylori* infection causes “the gastric ulceration”^24^ and, prolonged colonization leads site-specific diseases including dreadful gastric cancer.^25,26^ Human gastric biopsy samples were collected with known history of infection and inflammation. *H. pylori* was harvested from the gastric biopsy sample, cultivated, harvested, and characterized by invasive, noninvasive, and molecular techniques.

A group of 45 mice were housed in this group, where 25 mice were examined after 6 months while the remaining 20 after 12 months. Five mice died after 5 days of inoculation through oral gavages and were excluded from the study. Five new males were infected and added in the group to balance the number of animals in the 6 month time frame group. Four mice died after 47 weeks and their stomach were fixed and included in the 12 month group. Initial average weight was 20 ± 2.0 g, and no regular pattern of weight gain was observed. The weight of the animal was seen to depend on the severity of infection, and the maximum weight was found to be 34.0 ± 4.0 g and the minimum was 30.0 ± 2.0 g at the end of 6 months. After 6 months, few animals showed little increase in weight while others presented a weight loss, and the average weight at the time of dissection was 30.0 ± 3.0 g, except for two mice with significant tumors, pushing the average weight to 35 g. After selected time frames of inoculation, mice were dissected, blood was collected for serology, and the stomach for histology studies.

Gross anatomy study of the stomach showed an uneven surface with lesions at 6 months and abscess and protruded masses after 12 months (Figure 1e). Macroscopic studies showed the change in tissue morphology, abscess, hemorrhage, inflammation, and polypoid masses that turned in to severe pathological conditions and small polyp tumors (Figure 1f–h).

**Figure 3:**
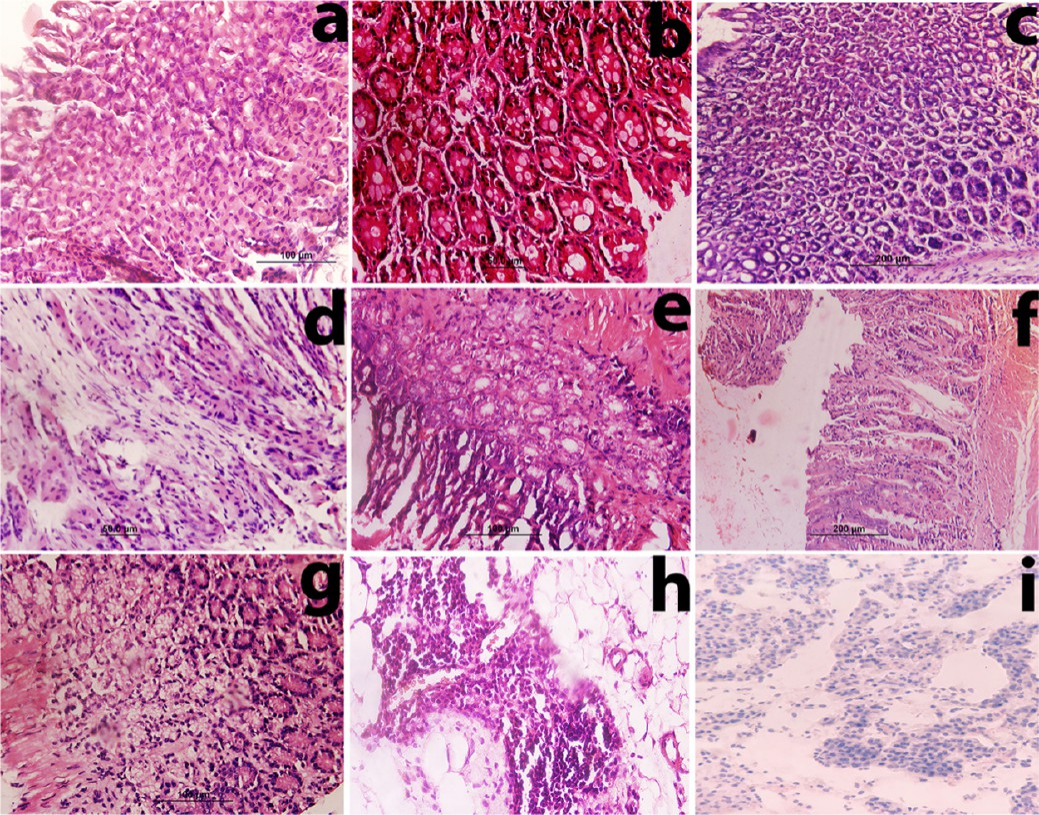
*H. pylori*-induced gastric carcinoma. Photomicrograph (H&E) reveals epithelial abnormalities, metaplasia, and adenocarcinoma. After 6 months: photomicrographs show (a) adenocarcinoma with abnormalities in parietal cells with variation in nucleus size, shape, number per cells, and fusion of cells, (b) complete metaplasia with epithelial abnormalities, and (c) adenocarcinoma with incomplete metaplasia, cell fusion, and structural abnormalities. After 12 months: photomicrographs show (d) morphological changes in gastric tissue due to GIST, (e) complete metaplasia with the influx of inflammatory cells and severe epithelial abnormalities, (f) invasive adenocarcinoma, (g) well differentiated adenocarcinoma, and (h,i) non-Hodgkin mantle cell lymphoma (histological sections show irregular nucleus distribution with the presence of pink histocytes).

Histological study of *H. pylori* infected mice for 6 months showed irregular-shaped follicular glands with hypertrophy, hyperplasia, epithelial abnormalities, fusion of cells, severe gastritis, atrophy, and in some cases metaplasia and dysplasia along with inflammatory response (Figure 3a–c). Inflammation also resulted in the loss of parietal cells that led to metaplasia and dysplasia in some mice. Epithelial abnormalities were also observed on histological sections such as erosion on the mucosal outer layer and severe architectural abnormalities of mucosal cells, inflammation, and cytoplasmic abnormalities due to atrophy. Polypoidal arrangement with dysplastic changes and adenocarcinoma having variation in nucleus size, shape, and number per cells were also observed in some cases. Two mice had adenocarcinoma with incomplete metaplasia, cell fusion, and structural abnormalities. All mice gave *H. pylori* positive results from the stomach at the time of dissection.

Histological sections of most of the mice after 12 months of infection demonstrated the presence of giant tumor cells and their fusion. Mice in this group suffered with carcinoma having variation in nucleus size, shape, and number per cells. GIST (Figure 3d) and polypoid adenocarcinoma were also observed in members of this group. A rare case of non-Hodgkin mantle lymphoma was also observed in two cases (Figure 3h,i). Complete metaplasia, which is the most common pathological condition associated with *H. pylori*, was also observed with the influx of inflammatory cells (Figure 3e) and epithelial abnormalities in induced gastric carcinoma after 12 months of infection. Invasive carcinoma (Figure 3f) and well differentiated (Figure 3g) and high-grade adenocarcinoma were also observed.

Table 1 shows the gastric pathology and incidence of gastric cancers in group 2 based on results of histological study (25% after 6 months and 55% after 12 months). At 12 months, almost all animals showed an influx of inflammatory cells and different types of pathological conditions including mixed types of carcinomas, metaplasia, and dysplasia.

*H. pylori* always counts as an associated reason for gastritis, and prolong gastritis leads to stomach cancer. *H. pylori* induction models not only help to study the host–microbe interaction but also provides insight into the mechanism of inflammatory carcinomas.^27^ Group 2 results are in agreement of previously published data of cancer induction by *H. pylori* with different incidence rates in different rodents.^28,29^

### 2.3 Group 3 (MNU + NaCl + *H. pylori-*Induced Cancer)

MNU is an established chemical carcinogen to develop gastric cancer in an animal model,^15^ with one limitation that models developed a lack of chronic inflammatory response and stomach pathology caused by *H. pylori* infections. In this research, therefore, a combination of *H. pylori* infection and MNU is used to get high incidence of gastric cancer as previously tested in Mongolian gerbils.^19^

A total of 60 mice were housed under this group, 30 were dissected and observed after 6 months and 30 after 12 months. A total of seven mice including three male and four female mice died before 20 weeks and were excluded from the study. Mice were kept under complete observation and their weight was measured on a regular basis. The initial average weight was 20.0 ± 2.0 g, and there was no specific pattern of weight gain in mice of this group. The average weight at the time of dissection was 26.0 ± 2.0 g at 6 months and 35.0 ± 3.0 g at 12 months. After the selected time frames, mice were dissected, blood was collected for serology, and stomach for histology and immunohistochemical studies.

**Figure 4:**
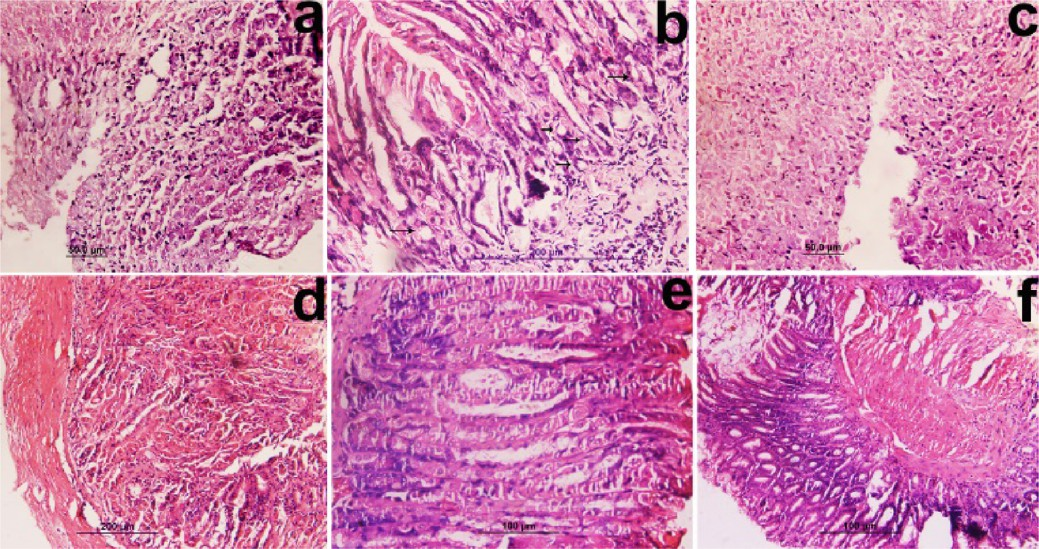
*H. pylori* + MNU + high salt diet-induced gastric carcinoma (after 6 months). The photomicrograph (H&E 40×) (a) reveals the morphological changes in gastric tissue due to GIST. Histological sections reveal spindle cells with marked atypia, hyperchromasia, and malignant gastrointestinal tumor, (b) represents the morphological changes due to the polypoidal carcinoma and formation of rings (indicated by arrows) due to signet ring cell carcinoma, (c) reveals GIST malignant gastrointestinal tumor with necrotic cells, (d) presents the poorly differentiated adenocarcinoma, (e) demonstrates high grade moderately differentiated carcinoma, and (f) reveals the morphological changes and the presence of squamous cell carcinoma.

Gross anatomy of the mouse stomach showed abnormal surface pathology with protruded masses, tumors, and inflammation. Surface morphology study showed an uneven surface, hemorrhage, inflammation, abscess, polyp, and small tumors (Figure 1i). Macroscopic examination showed uneven surfaces with lesion, abscess, forestomach sarcoma, several internal small polyp masses, and tumors (Figure 1j–l). Histological study (after 6 months) showed severe gastritis, atrophy, dysplasia along with inflammatory response, invasive carcinoma, GIST, polypoidal, signet ring cell carcinoma, metaplasia, squamous cell carcinoma, and adenocarcinoma. Microscopic studies of histological sections showed greater incidence of cancer than groups 1 and 2 with increased severity. Most of the histological studies showed a mixed type of cancer occurrence like polypoidal with signet ring cell carcinoma (Figure 4b). The histological section showed the influx of inflammatory cells in a mucus layer and moderately differentiated carcinoma (Figure 4e), poorly differentiated carcinoma (Figure 4d), diffused adenocarcinoma, GIST with spindles cells (Figure 4a), GIST with tumor necrotic cells (Figure 4c), and squamous cell carcinoma (Figure 4f).

**Figure 5:**
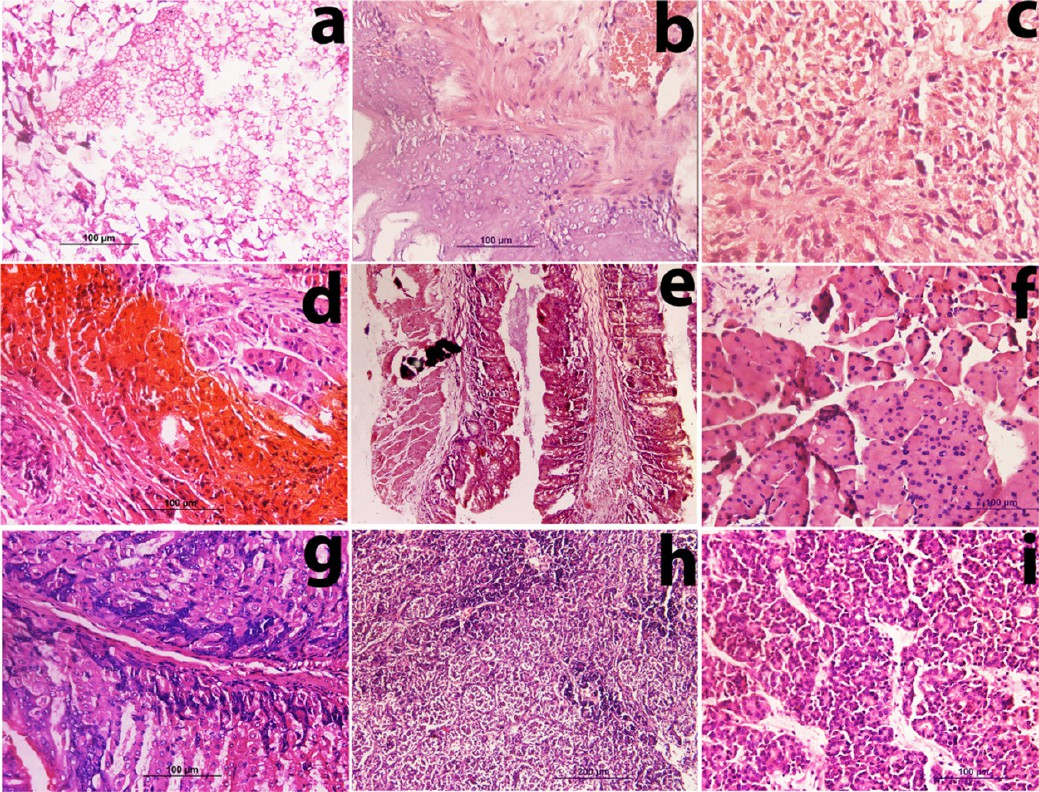
MNU+ high salt diet + *H. pylori-*induced gastric carcinoma (after 12 months). Photomicrographs (H&E) taken at 40× (a–d,f,g,h),10× (e), and 20× (i). (a) shows spread of carcinoma to submucosa (with floating tumor cell nests) and invasive adenocarcinoma. (b) shows squamous cell carcinoma. (c) shows poorly differentiated adenocarcinoma. (d) shows invasive adenocarcinoma with tumor hemorrhage. (e) shows adenocarcinoma with neuroendocrine carcinoma at the extreme left. (f) Tumor cells represent the characteristic features of neuroendocrine carcinoma with smaller salt and pepper nuclei. (g) shows high-grade adenocarcinoma with giant tumor cells. (h) shows the tumor section with poorly differentiated carcinoma. (i) presents the tumor section showing the lymphoma pattern.

**Figure 6:**
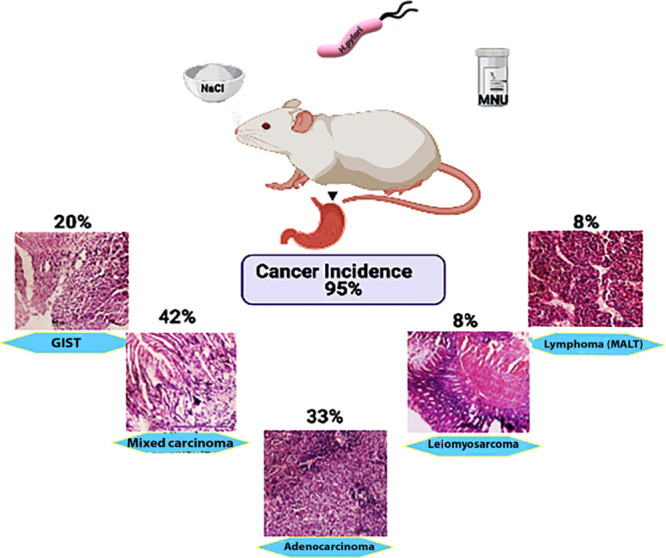
Schematic representation of gastric cancer incidence of group 3. Group 3 showed the maximum cancer incidence (95%) percentage with different types of gastric cancer.

Histological studies after 12 months showed severe inflammation of mucosa-associated lymphoid tissue (MALT) with the influx of neutrophils and monocytes in mucus cells and mucinous layers. Inflammation also resulted in the loss of parietal cells that led to dysplasia and adenocarcinoma. Microscopic studies revealed the presence of high-grade carcinoma of different types such as neuroendocrine carcinoma (Figure 5e,f), GIST with tumor nests (Figure 5a), signet ring cell carcinoma, squamous cell carcinoma (Figure 5b), necrotic tumor cells along with the lymphatic cells (Figure 5i), metaplasia, high-grade adenocarcinoma (Figure 5g), poorly differentiated adenocarcinoma (Figure 5c,h), and tumors (Figure 5d,i) including non-Hodgkin lymphoma. Severity of cancer in this group enhanced many-folds and showed to spread to all the layers of the stomach. Figure 5a shows invasive carcinoma’s spread to the submucosa and muscular layer with tumor nests floating. Table 1 shows the gastric pathology and incidence of gastric cancers that is 65% in group 3 after 6 months and 96% after 12 months, on the basis of histological study results. Inflammation was observed in most of the mice, and abscesses were seen in all the mice. Figure 6 shows the schematic presentation of cancer incidence and types of gastric cancer in group 3 after 12 months.

Cancer induction in group 3 (*H. pylori* + MNU + high dietary NaCl) showed the tremendous increase in the incidence of cancer (Figure 6). Histological studies showed existence of different forms of cancer with a high grade and severe abnormalities in cellular morphology. Most of the cancers were poorly differentiated carcinoma with greater incidence and severity in this group as compared to others even after 6 months. Herein, results clearly demonstrate that mouse models can be developed in a shorter time period by using the combination of *H. pylori* infection, chemical carcinogens (MNU), and effect-enhancing risk factors such as high concentration of NaCl. The severe stomach pathology conditions were observed in this group alongside of hallmarks of inflammation closer to the actual disease. The combination strategy of cancer induction is also reported in C57BL/6 J mice and scientists claimed a 100% cancer incidence rate in this species.^30^ In another study, the cancer incidence rate in C57BL/6 J mice with MNU and *H. pylori* was 68.8%.^31^

### 2.4 Targeted Diagnostic Probe Preparation and Characterization

**Figure 7:**
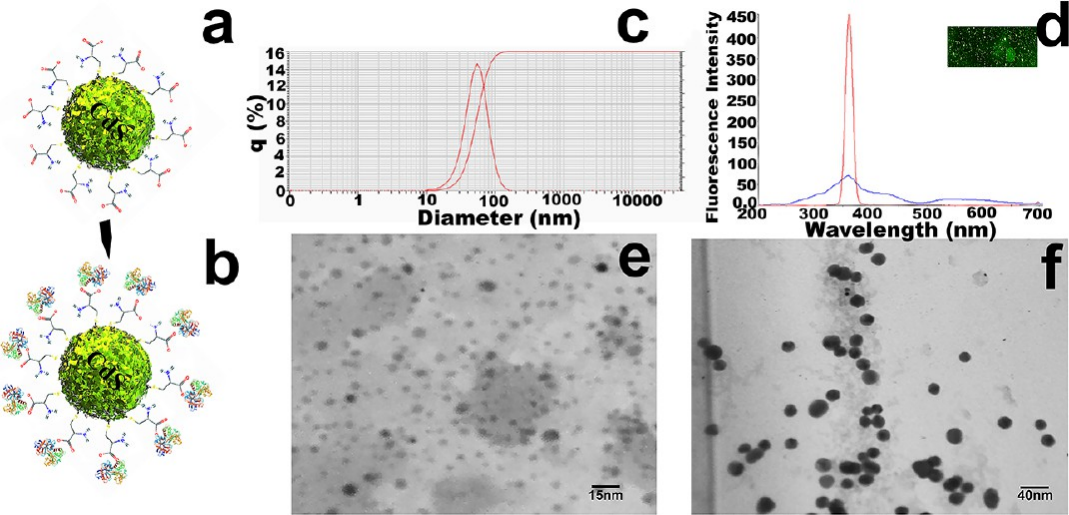
Schematic model presentation and characterization of the CdS QD-transferrin-targeted diagnostic probe. Figure (a) shows the schematic model of a cysteine-capped CdS QD and (b) shows the model of the targeted diagnostic probe. (c) DLS analysis shows that nanocomposites are in the size range of 20–100 nm with a mean size of 45 nm. (d) shows the fluorescence microscopy observation in the top right corner and excitation/emission spectra at 364.6 nm/366.1 nm. (e) Micrograph of cysteine-capped CdS QDs shows uniform, monodispersed, stable, and spherical nanoparticles of mean size 7 nm. (f) Micrograph shows that after conjugation of protein to cysteine-capped CdS QDs, the size of nanocomposites increased to 35 nm.

**Figure 8:**
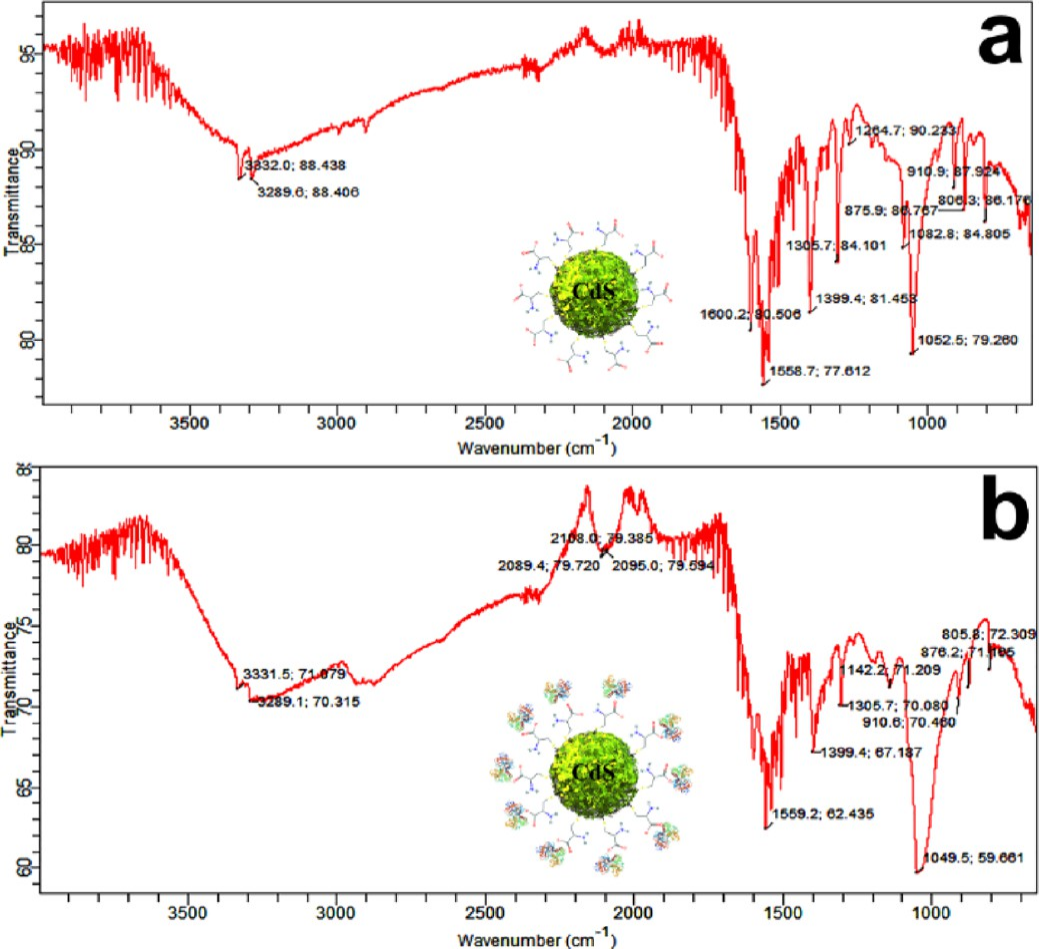
FTIR analysis. (a) FTIR spectrum of cysteine-capped CdS QDs shows the stretching and bending peaks of cysteine on the spine structure of the CdS QD. (b) FTIR spectrum of the diagnostic probe presents the successful conjugation of protein on the nanocarriers with alteration in the old peak position and introduction of new peaks.

QD nanoparticle size is very important for their optical properties and their applications as a biological marker. Surface chemistry can easily be studied through employing Fourier transform infrared (FTIR) spectroscopy and ensuring that surface chemistry has changed successfully after conjugation with linkers and the labels.^20^ The schematic model of cysteine-capped CdS QDs in Figure 7a,b shows the model of the targeted diagnostic probe after the conjugation of the transferrin protein amine group with the carboxylic group of cysteine. Modified QDs were characterized before conjugation (reported in the previous study with size below 7 nm^14^) and after conjugation with transferrin protein. Size of nanocomposites was analyzed by dynamic light scattering (DLS) and transmission electron microscopy (TEM), where DLS showed that the size of the targeted diagnostic probe was in the range of 20–100 nm with a mean size of 45 nm (Figure 7c). There was an increase of 25–30 nm after the conjugation of transferrin protein on cysteine-capped CdS QDs (Figure 7e–f). FTIR analysis was done in order to confirm the conjugation of transferrin on cysteine-capped CdS QDs for the preparation of the diagnostic probe. The FTIR spectrum of cysteine-capped CdS QDs (Figure 8a) showed the disappearance of the SH peak (2549.7 cm^–1^) that is utilized in the covalent attachment of cysteine sulfur with CdS sulfur with the introduction of new cysteine associated peaks: the most important one was seen at 3447.36 cm^–1^, which represented the N–H stretching of primary and secondary amines. The mild peaks of the N–H bending of amines were seen in the area defined by 1580–16 cm^–1^. The carboxylic group C=O stretching was seen at 1650.76 cm^–1^ and C–O stretching was observed at 1005.19 cm^–1^. Furthermore, attachment of transferrin protein was confirmed due to the alteration in the existing peak positions, intensities, and the introduction of new peaks (Figure 8b). Fluorescence of nanocomposites was observed by fluorescence microscopy and excitation and emission spectroscopy. Microscopy observation confirmed the sharp green fluorescence of nanocomposites under a blue excitation filter. Excitation and emission spectra showed maximum excitation at 364 nm and maximum emission at 366 nm (Figure 7d).

### 2.5 Transferrin Receptor Expression Studies by the Diagnostic Probe

**Figure 9:**
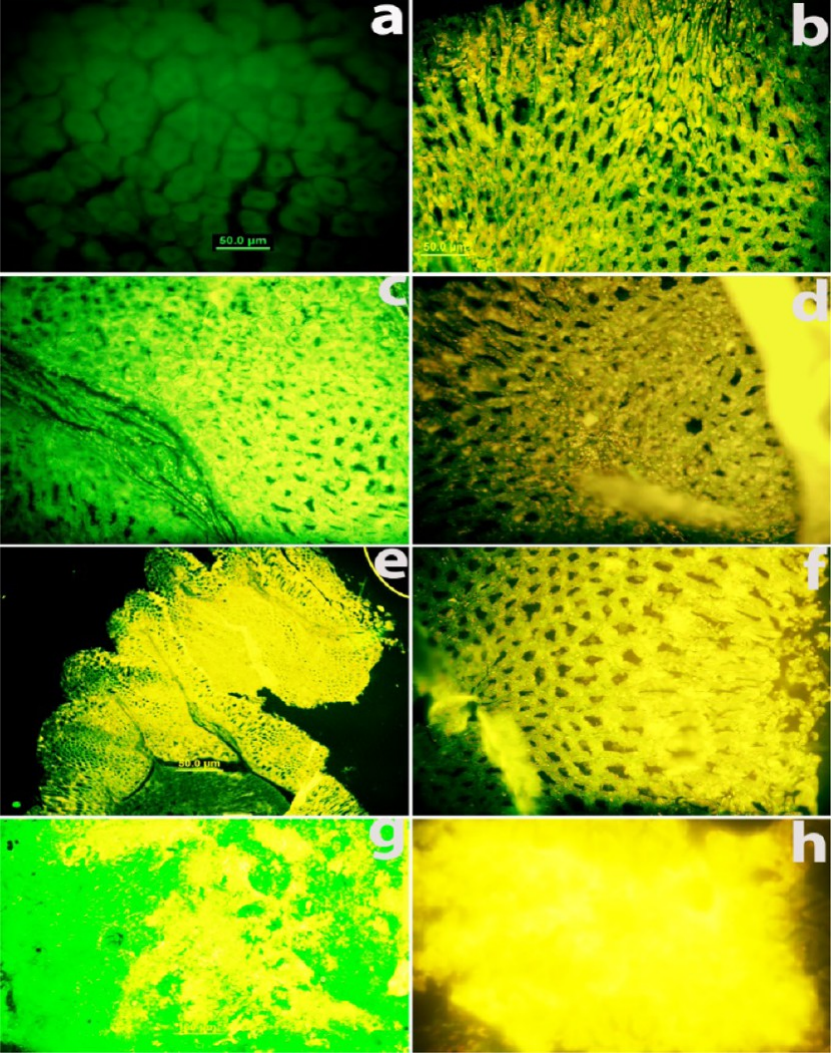
Transferrin receptor expression analysis on cancer-induced mice gastric tissue of all groups in comparison to the control CdS QD-conjugated antitransferrin binding to control gastric tissue and to gastric cancer/tumor tissues after tissue incubation with transferrin shows enhanced receptor expression in cancerous mice. (a) Control mice show background fluorescence, (b) transferrin receptor expression on group 1 mouse gastric tissue, (c) mice of group 2 show greater expression of the transferrin receptor but less than groups 1 and 3. (d) shows transferrin expression after 6 months of cancer-induction treatment in group 3 mouse gastric tissue. Group 3 mouse stomach tissues show the maximum expression of the transferrin receptor, many-fold enhanced than the normal tissue (e,f). The tumor section of group 3 also shows the many-fold enhanced expression of the transferrin receptor as shown in figures (g,h).

Gastric cancer/tumors tissues of all the groups were characterized for the expression of transferrin receptors. Tissues from infected mice were incubated in vitro with CdS-conjugated antitransferrin antibodies in order to confirm increased transferrin expression. Relative transferrin receptor expression of three groups and control is shown in Figure 9. Control mouse gastric tissue showed only limited background fluorescence while tissues from groups 1, 2, and 3 demonstrated fluorescence with many-fold enhanced intensity. Among the three groups, group 3 showed maximum fluorescence intensity that confirmed greater expression of transferrin receptors on gastric tissue of mice with maximum cancer incidence rates after exposing to *H. pylori* + MNU + high dietary salt NaCl for cancer induction. Results show that the incidence of cancer in group 3 was almost 100% This confirms that the transferrin receptor expression was greater in group 3 due to the severity and later stages of cancer. The mice of this group can be selected for targeted drug delivery experiments using transferrin as a targeting site.

These results are in line with the literature that reports tumor progression has high correlation with the increased expression of the transferrin receptor. Therefore, it could make for an attractive way to target cancer cells for drug delivery.^11^ Researchers synthesized the paclitaxel-loaded PLGA nanoparticles and conjugated them with transferrin to target human prostate cancer cells PC3. These results indicated the successful regression of tumor cancer cells in nude mice (Sahoo et al.^100^). Another study found that the targeted drug delivery to lung cancer cells by cationic polymer branched polyethyleneamine (bPEI) conjugated with the transferrin receptor-binding HAIYPRH peptide (HAI peptide). It provided a successful way of an efficient delivery of siRNA into TfR-overexpressing lung cancer cells. This targeted delivery resulted in efficient GAPDH gene knockdown.^32^ Another successful lung transferrin receptor-targeted strategy was reported by Guo et al. with transferrin-conjugated lipid-coated NPs (TFLP).^33,34^

### 2.6 Blood Assays (LFT and RFT)

**Figure 10:**
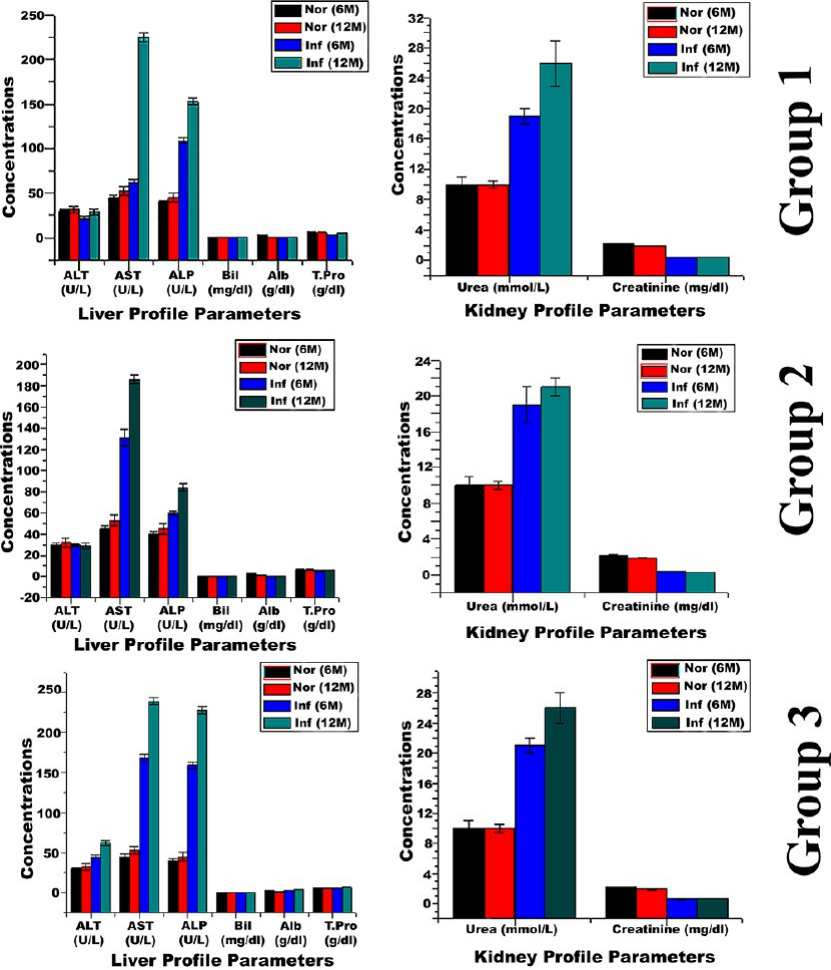
Statistical comparison of LFT and RFT of control and groups 1, 2, and 3 mice. Comparison of parameters of the liver profile shows higher levels of AST and ALP as compared to control and lower levels of albumin. Cancer-induced mice show higher urea levels as compared to control.

Liver and renal functional tests were also conducted in order to check the effect of different induction methods on these organs as shown in Figure 10. In liver function test (LFT) analysis of group 1, a minor elevation in aspartate aminotransferase (AST) and high increase in alkaline phosphatase (ALP) concentration were observed along with the lower level of albumin, while group 2 showed higher levels of AST and ALT after 6 months and a little higher level of these two enzymes after 12 months as compared to the control. In group 3, a higher level of AST and ALP concentration was observed along with lower levels of albumin. In renal function test (RFT) analysis, a higher level of urea with no effect on the creatinine level was observed in all groups (Figure 10).

## 3 Conclusions

Transferrin receptor expression was checked on gastric cancer/tumor tissues in relation to normal gastric tissues. Chemical treatment for cancer induction after *H. pylori* infection showed increased cancer incidence and increased transferrin receptor expression. This study describes a complete strategy for the development of a gastric cancer model in mouse and also confirmed the enhanced transferrin receptor expression on gastric cancer tissue. Transferrin receptors can be used as a site for targeted drug delivery to gastric and colon cancer. Studies are underway to target these receptors in order to find the best therapeutic strategy against gastric cancer.

## 4 Experimental Methods

### 4.1 Model Organisms and the Inducing Agents

BALB/c mice were used to develop models of gastric cancer by three methods. All animal studies were approved by the Animal Ethics Committee of the faculty of Life Sciences (Approval Diary Number D/3363-ACAD), University of the Punjab, Lahore, Pakistan. Methyl nitrosourea (MNU), pure food salt (NaCl), and the bacterial species *H. pylori* were used for cancer induction. *H. pylori* was isolated from human gastroendoscopy samples. It was characterized through 16S rRNA sequencing. MNU solution was prepared in sterilized tap water. *H. pylori* was cultured in brain heart infusion medium (BHI), and the culture was screened for CagA toxin, the positive culture for which was mixed and cultivated in BHI medium for 48 h under microaerophilic conditions at 37 °C and was used to induce cancer in mice. All the reagents were purchased from Sigma-Aldrich, UK.

### 4.2 Experimental Schedule

**Figure 11:**
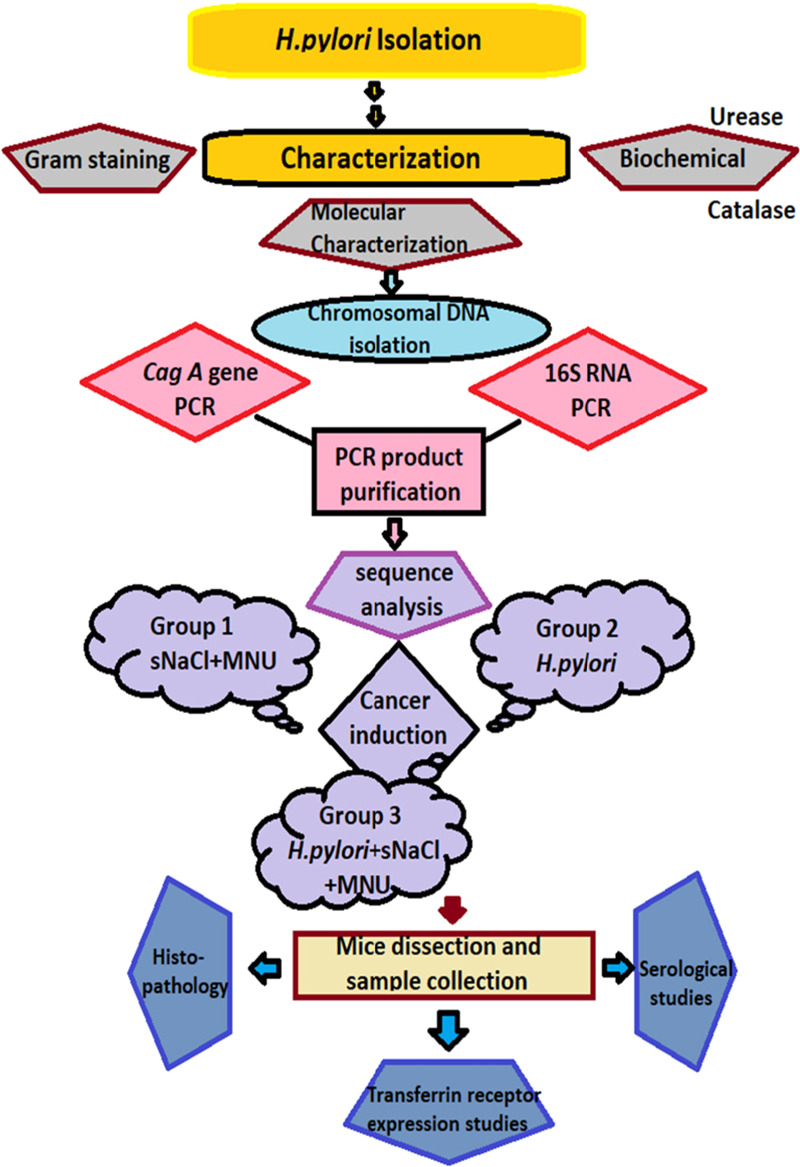
Flowchart of the methodology to develop the gastric cancer mouse model.

Animals from groups 1, 2, and 3 were killed after 6 months and 12 months from the starting time of induction. Control mice comprised males (*n* = 5) and females (*n* = 5) of 4–5 weeks old for each group. Males and females were kept in separate cages and fed on screened controlled diet and sterilized distilled water. Figure 11 shows the flowchart presentation of the conducted experiments to develop the gastric cancer mouse model.

#### 4.2.1 Group 1: MNU and NaCl-Induced Cancer

Group 1 contained four-week-old 25 male and 25 female mice. They were divided into two subgroups where the first group contained 20 mice (10 males/10 females). They were killed after 6 months, and the other group 30 mice (15 males/15 females) were killed after 12 months. During their life, mice were fed controlled diet and cancer was induced with MNU and NaCl; 250 mg of MNU was dissolved in 500 mL of sterilized tap water and 30% w/v NaCl was added. Supplemented water was used for the first 3 weeks of the treatment for cancer induction in light-shielded bottles to prevent photolysis. MNU solution was prepared fresh twice a week to replace the old solution. After 3 weeks, water was supplemented with MNU only and used for the rest of the period of induction. The animal weight was monitored throughout this period.

#### 4.2.2 Group 2: *H. pylori*-Induced Cancer

**Figure 12:**
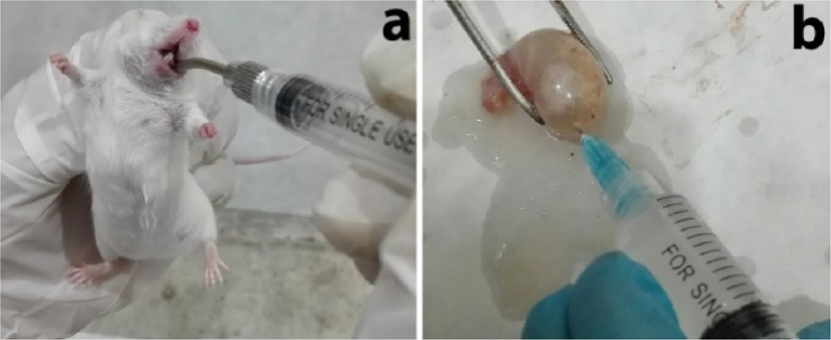
Oral dose administration and stomach washing; (a) shows oral dose (PO) administration to mouse through oral gavages and (b) shows the washing of the mouse stomach with saline solution.

Mice of group 2 were subdivided into two subgroups: 25 mice (10 males/15 females) were killed after 6 months, and 20 mice (10 males/10 females) were dissected after 12 months. *H. pylori* inoculation was used for cancer induction in group 2 mice. One hundred and twenty microliters of *H. pylori* suspension in BHI containing 1 × 10^9^ colony forming units (CFU)/mL were inoculated through orogastric gavages (intragastric tubes) once a day and thrice in the first week (Figure 12a). All mice were kept on an overnight fasting before inoculation with *H. pylori* and observed for half an hour after inoculation with orogastric gavages. After six inoculations with *H. pylori*, mice were fed screened pellet diet and filter sterilized tap water for the rest of their lives. Infected mice were housed in isolation to prevent the spread of infection among other groups. The animal weight was recorded throughout the period of treatment.

#### 4.2.3 Group 3: Cancer Induction by Combination of Salts and *H. pylori* Inoculation

Mice of group 3 were also subdivided into two subgroups: 30 mice (15 males/15 females) were killed after 6 months, and 30 mice (15 males/15 females) were dissected after 12 months. Mice of group 3 were treated in the same pattern as group 1 except inoculation with *H. pylori* (1 × 10^9^ colony forming units (CFU)/mL) was made into mice stomach through orogastric gavages thrice a week.

### 4.3 Mice Dissection and Sample Collection

At the time of dissection, the body weight of all mice was recorded. Before gastrectomy, blood from mice was collected in a vial and processed for serum isolation. Serum was stored at −20 °C. After dissection of mice, the stomach was removed and washed with 0.85% NaCl solution with a syringe (Figure 12b).

The stomach was incised along the greater curvature and the gross anatomy of gastric tissue was examined. The stomach was fixed in lysine-periodate-paraformaldehyde (PLP) fixative for histopathological studies.

### 4.4 Histopathological Studies

PLP-fixed tissues were dehydrated in an ascending order of ethanol (50, 70, 80, 90, and 100%) concentration for 6 h to overnight at each step. The fixed tissue was filtered with ethanol:xylene (1:1 ratio) and then with 100% xylene. Tissues were embedded in a paraffin block under the standard protocol of AMBR lab^35^ and incubated at 50 °C for 3 h. Paraffin-embedded tissues were mounted on the specimen disk of a microtome and 8 μm sections were cut. The sections were placed on albumin-coated slides and processed for hematoxylin and eosin (H&E) staining to study the tissue under a microscope.

Tissue sections were rehydrated in a descending order of ethanol (100, 90, 80, and 70%) concentration and stained with hematoxylin stain for 8 min. Tissues were washed gently in running tap water and were differentiated with 1% acid alcohol (1% HCl in 70% ethanol), followed by staining with eosin, and a final wash to remove the unbound stain. Tissues were dehydrated again in an ascending order of alcohol and washed off with xylene. Tissue sections were mounted with a mounting medium and observed in a bright field microscope (Olympus BX51).^36^

### 4.5 Targeted Diagnostic Probe Preparation

Cysteine-capped cadmium sulfide QDs were synthesized in the presence of taurine (antioxidant), as described in the previous study. Transferrin was conjugated with cysteine-capped CdS QDs by the carbodiimide method and used as a cancer-targeting probe. Briefly, 0.01 g of nanocarriers (CdS QDs in 1 mL of deionized water, pH:8) was mixed with 1 mL of EDC (20 mg mL^–1^ of deionized water) and pH 6.4 was adjusted with 1 M HCl. After the incubation of 30 min with continuous shaking, 1 mL of carbodiimide (10 mg mL^–1^ of deionized water) was added again with 100 μg of purified transferrin. The reaction mixture was incubated in the dark for further 2 h at 37 °C with continuous shaking at 100 rpm. After incubation, modified QDs were isolated by centrifugation at 14000*g* for 10 min at 4 °C and washed twice with PBS. The washed QDs were suspended in 1× TBS buffer for further use and stored at −20 °C.

### 4.6 Characterization of the Targeted Probe

Samples were prepared according to the given instructions of the respective instrumental manual for the characterization of synthesized cysteine-capped CdS. All the solutions were degassed under vacuum before proceeding for further characterization using different techniques. Excitation and emission spectra were obtained on a fluorescence spectrophotometer (PerkinElmer fluorescence spectrophotometer, LS45) by exciting in the range of 359–364 nm, and the maximum emission intensity and wavelength were recorded. Fluorescence of bare CdS and Cys-CdS QDs was also observed under different filters of a fluorescence microscope (Olympus BX51); UV (excitation 350 nm with blue emission 450 nm), blue (excitation 450 nm with green emission 550 nm), and green filters of a fluorescence microscope (excitation 550 nm with deep red emission 690 nm). Surface chemistry of cysteine-capped CdS and transferrin conjugation were studied by FTIR spectroscopy (FTIR spectrometer, Agilent Technology Cary 630). The size and shape were studied by TEM (JEOL JEM-1010) and a DLS analyzer (HORIBA Scientific, nanoPartica SZ-100) under standard conditions.

### 4.7 Transferrin Receptor Studies

Dissected stomachs from all mouse group were fixed in a PLP fixative and studied for the transferrin expression. Tissues were dehydrated in a sucrose gradient and embedded in the OCT compound. Sections of 6–7 μm were cut on a cryostat and placed on albumin-coated slides and blocked with 5% skimmed milk. Sections were incubated with modified QDs for 1 h in a humidified chamber at 37 °C and washed with 1× TBS buffer. Tissue sections were observed under a blue excitation filter through a fluorescence microscope.

### 4.8 Serological Studies

Stored mouse sera were thawed and analyzed by using a commercial kit HumaLyzer for liver and renal function tests within 2 days. ALP, AST, alanine aminotransferase (AAT), bilirubin, albumin, and total proteins were the parameters in LFTs, whereas creatinine and urea were determined in RFTs.

### 4.9 Statistical Analysis

Data were analyzed by one way analysis of variance and presented as a mean with standard deviation. SPSS version 26 was used for analysis including mean ± SD and result significance was declared at *p* < 0.05. A paired *t*-test was also applied to check the significance between groups at specific conditions and unpaired to check significance of values within groups at two different times. OriginPro 8 was used for plotting graphs.
